# Effect of Red Clover Isoflavones over Skin, Appendages, and Mucosal Status in Postmenopausal Women

**DOI:** 10.1155/2011/949302

**Published:** 2011-11-01

**Authors:** Markus Lipovac, Peter Chedraui, Christine Gruenhut, Anca Gocan, Christine Kurz, Benedikt Neuber, Martin Imhof

**Affiliations:** ^1^Division of Obstetrics and Gynecology, General Public Teaching Hospital Korneuburg, 2100 Korneuburg, Austria; ^2^Instituto para la Salud de la Mujer, Facultad de Ciencias Médicas, Universidad Católica de Santiago Guayaquil, 196 Guayaquil, Ecuador; ^3^Study Center Med XIX, Grinzingerstr 83, 1190 Vienna, Austria; ^4^Division of Obstetrics and Gynecology, Medical University of Vienna, 1090 Vienna, Austria

## Abstract

* Objective*. Evaluate in postmenopausal women the effect of red clover extract (RCE) isoflavones over subjective status of skin, appendages, and several mucosal sites. *Method*. Postmenopausal women (*n* = 109) were randomly assigned to receive either two daily capsules of the active compound (80 mg RCE, Group A) or placebo of equal appearance (Group B) for a 90-day period. After a washout period of 7 days, medication was crossed over and taken for 90 days more. Subjective improvement of skin, appendages, and several mucosal site status was assessed for each studied group at 90 and 187 days using a visual analogue scale (VAS). In addition, libido, tiredness, and urinary, sleep, and mood complaints were also evaluated. *Results*. Women after RCE intervention (both groups) reported better subjective improvement of scalp hair and skin status, libido, mood, sleep, and tiredness. Improvement of urinary complaints, nail, body hair, and mucosa (oral, nasal, and ocular) status did not differ between treatment phases (intra- and intergroup). Overall satisfaction with treatment was reported higher after RCE intervention (both groups) as compared to placebo. *Conclusion*. RCE supplementation exerted a subject improvement of scalp hair and skin status as well as libido, mood, sleep, and tiredness in postmenopausal women.

## 1. Introduction

As a result of increased life expectancy, nowadays, women spend more than one third of their lives in a state of estrogen deprivation which in turn leads to a number of significant long-term changes [1]. Indeed, two out of three women in the menopausal transition present different complaints [2]. Increased osteoporosis and cardiovascular risk, vasomotor episodes, and sleep disturbances have been the main research topics and focus of treatment. Others aspects such as urinary complaints, loss of libido, and changes in hair, nail, skin, or mucosal status have often been disregarded (neglected) by researchers and health care providers but not by women themselves.

Hormone therapy (HT), using different estrogenic compounds, has shown its effectiveness over a number of these complaints; nevertheless, current HT use has become controversial due to suspected increased risk of breast cancer and cardiovascular disease [3]. Furthermore, 10% of western women display conditions that contraindicate HT use: history of estrogen-dependent malignancy, liver disease, thromboembolic disorders, and severe migraine [4]. Under this scenario, the use of alternatives to HT such as botanical and dietary supplements (i.e., phytoestrogens) has increased for the management of menopausal complaints [2]. Beside coumestrols and flavonoids, isoflavonoids are the main active substances of “phytoestrogens” [5]. Epidemiological and clinical research has shown the positive effects of soy isoflavone consumption over bone [6–8] and the risk of developing several female cancers [9–14]. Reports indicate that isoflavones exhibit estrogenic activity through the activation of estrogen receptors (ERs) and also antiestrogenic activity by competitive binding to the ER and estradiol (E_2_) inhibition. Moreover, they have a significantly higher affinity for the ER-beta than ER-alpha [15]. 

Trifolium pratense or red clover (RC) is a perennial herb growing in all temperate and subtropical areas around the world. In several cultures, it is used as traditional medicine [16]. Besides its daidzein and genistein content, unlike soy, RC displays a high content of methylized precursors: biochanin A and formononetin [5]. After several years of clinical research with RC, there is evidence supporting their positive effects in climacteric women in terms of vasomotor symptoms [17], bone mineral density [18], mood [19], vaginal and sexual health [20, 21], and serum lipids [22]. Despite this, RC data on skin, appendages, including mucosal sites, are scarce or lacking. Hence, the aim of the present paper was to evaluate in postmenopausal women the effect of red clover extract (RCE) isoflavones over subjective status of skin, appendages, and several mucosal sites. In addition, libido, tiredness, and urinary, sleep, and mood complaints were also evaluated.

## 2. Methods

### 2.1. Study Design and Participants

A prospective randomized, double-blind, placebo-controlled trial was carried out from May 2003 to November 2004 at the Study Center Med XIX and the Department of Gynecological Endocrinology and Reproductive Medicine, General Hospital of the Medical University of Vienna, Vienna, Austria. Study protocol was approved by the Ethics Committee of the above-mentioned university and aimed at evaluating the effect of red clover isoflavone supplementation in postmenopausal women, with several proposed targets: vasomotor and general menopausal symptoms, selected sex hormones and endometrium [1], and depressive/anxiety symptoms [19]. The present paper specifically presents data on the effect of RCE over subject skin, appendages, and mucosal status in addition to libido, tiredness and urinary, sleep, and mood complaints. 

 Postmenopausal women (amenorrhea >12 months), 40 years or older with moderate-to-severe menopausal symptoms (Kupperman index ≥15) were recruited from the daily routine of the Menopause Ambulance of the General Hospital and the Study Center Med XIX as previously described [1]. Those with a positive pregnancy test, nonwillingness to adhere to the control dates and take the prescribed preparations, on hormonal therapy (HT), or with known isoflavone hypersensitivity were excluded. A baseline FSH >35 mlU/mL was confirmatory of postmenopausal status [1]. 

After being informed about the research (aims and method) and providing written consent, participants were randomly assigned to receive either two capsules of the active RCE compound (80 mg red clover isoflavones, Group A) or placebo of equal appearance (Group B) for a 90-day period. After a 7-day washout period, subjects switched to receive the opposite treatment for another 90 days. Additional examinations comprised anamnesis, medication anamnesis, and height, weight, and blood pressure determinations at proposed intervals [1]. Body mass index (BMI) was calculated as weight (kg)/squared height (m) [20].

### 2.2. Assessment of Studied Parameters

Studied variables related to subjective skin, appendages, and mucosal status and libido, tiredness, and urinary, sleep, and mood complaints were measured for each studied group at 90 and 187 days using a visual analog scale (VAS) scored from 0 to 100. Scores were indicative of subjective percent improvement of the symptom or condition.

### 2.3. Preparations

RCE capsules contained a standardized content of 40 mg aglyconic isoflavones in form of biochanin A, formononetin, genistein, and daidzein. RCE and placebo capsules (90 mg lactose) were of identical design, packed in opaque containers, labeled as A or B, and blinded to investigators and participants until the end of the trial after which the code was broken.

### 2.4. Statistical Analysis

Statistical analysis was performed on an intention-to-treat basis using SPSS software package (Version 19.0 for Windows, SPSS Inc., Chicago, Ill, USA). Data are presented as means (standard deviations), medians (interquartile ranges [IQR]), and percentages. The Shapiro-Wilk test was used to determine the normality of data distribution. Differences between groups were analyzed with the Mann-Whitney (continuous non parametric data) or the Chi-square test (percentages). Changes after each treatment phase within studied groups were assessed using the Wilcoxon rank test. A *P* value < 0.05 was considered as statistically significant. Sample size calculation was primarily based on the assumption that hot flush frequency would be reduced 50% in the RCE group (15% in the placebo group). Hence, a sample size of 49 individuals per group was calculated in order to achieve an 80% power at a two-sided alpha level of 0.05.

## 3. Results

During the study period, a total of 113 women consented to participate. Fifty-three were randomized to group A and sixty to group B. Four women started HT and were excluded. Thus, data of 109 women who completed treatment (Group A: 50 and Group B: 59) was used for analysis. No significant differences were observed between study groups regarding basal characteristics (Table 1). Hysterectomy was in all cases due to a benign cause (i.e., uterine fibroids), and former HT users had stopped intake at least 6 months prior to recruitment with a maximum previous intake of 7.5 years. No side effects were encountered after treatment with the active compound or the placebo group.

Women in both studied groups reported a higher VAS subjective percent improvement of scalp hair and skin status, libido, mood, sleep, and tiredness after RCE intervention as compared to placebo. In general, reported improvement over urinary complaints, nail, body hair, and mucosa (oral, nasal, and ocular) status did not differ between treatment phases (intra- and intergroup). Women in both groups reported higher subjective improvement of digestive complaints after placebo. Overall satisfaction with treatment was reported higher after RCE intervention in both assigned groups as compared to placebo (Table 2). 

## 4. Discussion

In the past decade, there has been an increasing trend toward individualizing treatment options for the menopause with a interesting focus on alternatives to estrogens [23–25]. This situation has occurred due to risk-benefit issues raised after the publication of the WHI results [23]. Tendency seems to be more pronounced among those with contraindications or with high-risk situations. Among available alternative treatments, one can mention phytoestrogens, plant-derived molecules, mainly represented by isoflavones, that display estrogenic like effects [26–28]. Although less potent than conventional estrogenic compounds, their selective beta-estrogenic receptor-binding properties allow beneficial effects on specific organs or systems [28]. Interest in red clover isoflavones (a kind of phytoestrogen) has equally grown among physicians and women with reports evidencing positive effects over menopausal symptoms [20, 29], vaginal health [21], serum lipids [30], and a promising safety profile [1]. However, to date, few or no studies using isoflavones have reported effects on skin, appendages, and mucosal status. 

Compared to the effect of estrogens on hair thickness or loss, effects on growth have been poorly investigated [31]. Estrogens seem to prolong the anagen cycle of the hair follicle [32]. Although hair follicle is an ER2 (beta) target [33], growth could predominantly be regulated by the insulin like growth factor 1 (IGF-1) [34] through crosstalk with the ER1 (alpha) [35]. A recently published mouse model trial showed isoflavone-induced IGF-1 increase in hair follicle dermal papilla cells, and thereby increased hair growth [36]. The present study found that women reported better subjective improvement of scalp hair and skin status after RCE intervention as compared to placebo. Reported improvement of nail, body hair (less growth), or mucosal status did not statistically differ among studied phases (intra- and intergroup). Regarding the latter, important to mention is that although the pathophysiology of the menopausal “burning mouth syndrome” is still unclear [37], HT use may improve this condition [38–41]. Although a Finnish group detected ER2 as the predominant estrogenic receptor in oral mucosa and salivary gland, they also pointed out that there are many other potent regulatory mechanisms possibly involved in the regulation of mucosal status [41]. Nasal mucosa expresses ER2 yet no ER1 or progesterone receptor (PR). ER2 count decreases with age (men and women) at this site and seems directly associated to quality of life [42]. Women of our series reported no subjective improvement on nasal mucosal status (dryness and fissuring). 

In the present study, women of both groups reported better subjective improvement of skin condition (better texture, moisture, and overall condition) after RCE phase as compared to placebo. ER1 and ER2 have been shown in certain skin layers but expressed differently according to the skin region. Lower risk for malignant skin changes in women is partly due to the higher estrogen status than in men [43]. It has been reported that HT clinically improves skin elasticity, thickness, and hydration [44] and reduces shrinks [45]. Surprisingly, our results seem to favor the fact that not only ER1 yet also ER2 can be expressed in the epidermis of different anatomical sites [31]. Despite this, we must bear in mind isoflavones' SERM profile displaying also anti-inflammatory [46], UV protective [47], and wound healing [48] potentials.

No subjective improvement over urinary complaints were found in our study despite the fact that isoflavones have shown positive effects on the urethral sphincter mechanism in the animal model [49] and in patients with prostate carcinoma [50]. The latter clinical study [50], however, used a rather high isoflavone dose (200 mg/day for 6 months). This dosage and duration are still uncommon in clinical menopausal isoflavone trials.

Better subject improvement of libido, mood, sleep, and tiredness was also evident in the present series after RCE supplementation as compared to placebo (both groups). Chedraui et al. [21] and Hidalgo et al. [20] have demonstrated improvement of libido and vaginal complaints after RCE treatment. Contrarily, del Giorno [51] could not find libido improvement after using 40 mg/day RCE dose which is lower than that used in our trial. We have previously reported that RCE supplementation improved anxiety and depressive symptoms in postmenopausal women [19], a fact that supports our present data. 

Finally, the authors acknowledge the limitations of analyzing subjective data obtained with a VAS and the lack of VAS-baseline values for the studied parameters in order to perform comparisons. However, VAS baseline assessment was not carried due to the fact that many women could not recall exactly when complaints started. Despite this, to date, the effect of RCE on skin, appendages and, mucosal status has not been reported, and this may be seen as a potential added value of the present study. However, more research is required in order to support our preliminary findings on these aspects. 

In conclusion, RCE supplementation exerted a subject improvement of scalp hair and skin status as well as libido, mood, sleep, and tiredness in postmenopausal women.

##  Conflict of Interests

The authors declare that they have no conflict of interests.

## Figures and Tables

**Table 1:** Demographic data of studied women at baseline*.

	All (*n* = 109)	Group A (*n* = 50)	Group B (*n* = 59)
Age (years)	53.5 ± 7.1	54.5 ± 6.2	53.7 ± 7.8
BMI (Kg/m^2^)	24.7 ± 3.9	24.5 ± 3.9	24.9 ± 3.9
Hysterectomy (%)	17 (15.6)	9 (18.0)	8 (13.6)
Former HT use (%)	64 (58.7)	29 (58.0)	35 (59.3)

*Data are presented as mean ± standard deviations or percentages (*n*, %); BMI: body mass index; HT: hormone therapy; Group A: RCE; Group B: placebo.

**Table 2:** Subjective symptom or condition improvement after treatment as assessed with the VAS.

Studied parameters	Group A		Group B	
	After RCE	After placebo	After placebo	After RCE
Scalp hair (better texture, less fragility, and overall condition)	7.3 ± 16.6^†^ [0, 0]	4.2 ± 13.9 [0, 0]	0.2 ± 0.9 [0, 0]	6.3 ± 13.9* [0, 0]

Body hair (less growth)	6.4 ± 16.5 [0, 0]	2.6 ± 10.9* [0, 0]	1.0 ± 3.4 [0, 0]	2.2 ± 6.2 [0, 0]

Skin condition (better texture, more moisture, and better overall condition)	18.6 ± 20.5^†^ [15, 32]	6.2 ± 16.2* [0, 0]	5.0 ± 11.0 [0, 5]	17.7 ± 21.2^∗†^ [5, 35.0]

Ocular complaint (dryness and burning)	7.8 ± 18.0^†^ [0, 7]	5.3 ± 15.1 [0, 0]	2.2 ± 9.5 [0, 0]	6.0 ± 13.7 [0, 1.3]

Oral mucosa complaint (dryness and burning)	3.9 ± 14.0 [0, 0]	3.3 ± 13.0 [0, 0]	1.8 ± 5.2 [0, 0]	2.8 ± 9.7 [0, 0]

Nasal mucosa complaint (dryness and fissuring)	4.2 ± 15.3 [0, 0]	2.6 ± 9.1 [0, 0]	1.6 ± 5.3 [0, 0]	6.2 ± 16.1* [0, 1.3]

Nails condition (change in thickness and stability)	5.6 ± 16.2 [0, 0]	6.2 ± 16.7 [0, 0]	3.3 ± 10.0 [0, 1.3]	10.8 ± 19.3* [0, 11.3]

Digestive complaints (diarrhea and constipation)	6.0 ± 16.1^†^ [0, 0]	14.4 ± 19.4* [0, 32.0]	19.5 ± 26.0 [6, 31.3]	11.9 ± 26.4* [0, 10.8]

Libido	18.0 ± 16.7^†^ [17, 30]	4.9 ± 14.3* [0, 0]	5.0 ± 12.4 [0, 1.3]	17.8 ± 20.9^∗†^ [10, 30]

Urinary complaint (incontinence and dysuria)	5.2 ± 18.1 [0, 0]	5.4 ± 17.3 [0, 0]	3.0 ± 13.7 [0, 0]	4.7 ± 15.0 [0, 0]

Mood complaint	68.5 ± 33.6^†^ [80, 48]	15.0 ± 25.2* [0, 25.3]	7.7 ± 19.2 [0, 0.8]	65.8 ± 37.8^∗†^ [78.5, 70.5]

Sleeping complaint	73.5 ± 33.4^†^ [90, 45]	16.2 ± 25.7* [0, 25]	9.8 ± 23.0 [0, 7]	70.6 ± 3.5^∗†^ [81.5, 62.2]

Tiredness	61.7 ± 45.8^†^ [96, 100]	16.1 ± 24.5* [0, 26.3]	8.3 ± 22.7 [0, 0]	56.1 ± 47.7^∗†^ [92.0, 100]

Overall satisfaction with treatment	87.3 ± 26.6^†^ [100, 12.5]	29.8 ± 31.4* [30, 50]	14.7 ± 26.8 [0, 19]	81.7 ± 26.8^∗†^ [100, 43.7]

Values are expressed as percent improvement and presented as mean ± standard deviations [median, interquartile range]; *P < 0.05 when comparing phases in same group using Wilcoxon rank test; ^†^ *P* < 0.05 as compared to placebo phase of the contrary group using the Mann-Whitney test.
